# Evidence for host effect on the intestinal microbiota of whitefish (*Coregonus* sp.) species pairs and their hybrids

**DOI:** 10.1002/ece3.5676

**Published:** 2019-10-02

**Authors:** Maelle Sevellec, Martin Laporte, Alex Bernatchez, Nicolas Derome, Louis Bernatchez

**Affiliations:** ^1^ Institut de Biologie Intégrative et des Systèmes (IBIS) Pavillon Charles‐Eugène‐Marchand Université Laval Québec QC Canada

**Keywords:** captive whitefish, intestinal microbiota, speciation, wild whitefish

## Abstract

Investigating relationships between microbiota and their host is essential toward a full understanding of how animal adapt to their environment. Lake Whitefish offers a powerful system to investigate processes of adaptive divergence where the dwarf, limnetic species evolved repeatedly from the normal, benthic species. We compared the transient intestinal microbiota between both species from the wild and in controlled conditions, including their reciprocal hybrids. We sequenced the 16s rRNA gene V3‐V4 regions to (a) test for parallelism in the transient intestinal microbiota among sympatric pairs, (b) test for transient intestinal microbiota differences among dwarf, normal, and hybrids reared under identical conditions, and (c) compare intestinal microbiota between wild and captive whitefish. A significant host effect on microbiota taxonomic composition was observed when all lakes were analyzed together and in three of the five species pairs. In captive whitefish, host effect was also significant. Microbiota of both reciprocal hybrids fell outside of that observed in the parental forms. Six genera formed a bacterial core which was present in captive and wild whitefish, suggesting a horizontal microbiota transmission. Altogether, our results complex interactions among the host, the microbiota, and the environment, and we propose that these interactions define three distinct evolutionary paths of the intestinal microbiota.

## Graphical abstract

Investigating the holobiont system is essential toward a full understanding of how animal adapt to their environment and, ultimately, speciation. We compared and sequenced the transient intestinal microbiota among dwarf and normal forms as well as their reciprocal hybrids of Lake Whitefish from the wild and in controlled conditions. Our results suggest complex interactions among the host, the microbiota, and the environment.

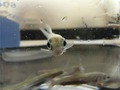

## 1 INTRODUCTION

Woese (1998) referred the Earth as a microbial planet, where macro‐organisms are recent additions. Indeed, an increasing number of studies have highlighted the substantial impact of microbiota on their host genes (Hooper et al., 2001; Rawls, Samuel, & Gordon, 2004) and that microbiota may be transmitted across generations in both animals and plants (Rosenberg & Zilber, 2016). In fishes in particular, the mother allocates antimicrobial compounds to the eggs before spawning (Hanif, Bakopoulos, & Dimitriadis, 2004; Wilkins, Rogivue, Fumagalli, & Wedekind, 2015). This maternal selection of bacteria influences the first bacteria that will be in contact with the sterile larvae during hatching (Llewellyn, Boutin, Hoseinifar, & Derome, 2014). Clearly then, a holistic understanding of macro‐organisms biodiversity requires the investigation of their association with microbiota and their co‐evolution (Miller, Svanbäck, & Bohannan, 2018).

The hologenome concept stipulates that the genome of the host and the microbiome (i.e., sum of the genetic information of the microbiota) act in consortium as a unique biological entity, that is, the holobiont (Rosenberg & Zilber, 2013). Consequently, the microbiota may be involved in host reproductive isolation, either in the form of a prezygotic barrier by influencing the host's mate choice by chemosensory signals (Brucker & Bordenstein, 2012; Damodaram, Ayyasamy, & Kempraj, 2016; Shropshire & Bordenstein, 2016) or in the form of a postzygotic barrier by producing genome and microbiome incompatibilities in hybrids, in accordance with the Bateson, Dobzhansky, and Muller model of genetic incompatibilities (Brucker & Bordenstein, 2012; Dobzhansky, 1937; Muller, 1942). Because the bacterial community of the gastrointestinal tract is implicated in many critical functions essential for development and immune responses, the intestinal microbiota could play an important role on its host's adaptive potential (Alberdi, Aizpurua, Bohmann, Zepeda‐Mendoza, & Gilbert, 2016; Macke, Tasiemski, Massol, Callens, & Decaestecker, 2017; Rosenberg & Zilber, 2013).

Fishes as a group comprise the greatest taxonomic diversity of vertebrates and a major food resource for human populations (Béné et al., 2015; Nelson, 2006), yet little is known about the relationship with their microbiota compared with the already well‐characterized mammals and insect microbiota (Clements, Angert, Montgomery, & Choat, 2014). The Lake Whitefish (*Coregonus clupeaformis*) is a well‐studied system that represents a continuum in the early stage of speciation where sympatric species pairs of dwarf and normal species evolved independently in several lakes in northeastern North America (Bernatchez et al., 2010; Rougeux, Bernatchez, & Gagnaire, 2017). The normal species is specialized for using the trophic benthic niche, feeding on diverse prey as zoobenthos and molluscs. It is characterized by rapid growth, late sexual maturity, and a long lifespan (Bodaly, 1979; Landry & Bernatchez, 2010). In contrast, the dwarf whitefish is a limnetic specialist which feeds almost exclusively on zooplankton and is characterized by slower growth, early sexual maturation, and shorter lifespan compared with the normal species. Previous transcriptomic studies revealed overexpression of genes implicated with survival functions (e.g., enhanced swimming performance for predator avoidance, detoxification) in dwarf whitefish, whereas normal whitefish show overexpression of genes associated with growth functions (Bernatchez et al., 2010; StCyr, Derome, & Bernatchez, 2008). Moreover, many other physiological, morphological, and behavioral traits display parallel differences among these two whitefish species that correspond to their respective trophic specialization (Bernatchez et al., 2010; Dalziel, Laporte, Guderley, & Bernatchez, 2017; Dalziel, Laporte, Rougeux, Guderley, & Bernatchez, 2016; Dalziel, Martin, Laporte, Guderley, & Bernatchez, 2015; Gagnaire, Normandeau, Pavey, & Bernatchez, 2013; Jeukens, Bittner, Knudsen, & Bernatchez, 2009; Laporte, Dalziel, Martin, & Bernatchez, 2016; Laporte et al., 2015). Thus, the recent speciation and the clear trophic segregation make the whitefish species pair an excellent model to study the role of intestinal microbiota in the context of ecological speciation.

Two previous studies documented the variation in two microbial niches in Lake Whitefish species pairs: the kidney and the intestinal adherent communities (Sevellec, Derome, & Bernatchez, 2018; Sevellec et al., 2014). Although we observed parallel patterns of differentiation between normal and dwarf species in the bacterial kidney communities, no clear evidence for parallelism was observed in the adherent intestinal microbiota. However, the water bacterial community was distinct from the adherent intestinal microbiota, suggesting an intrinsic properties of the host microbiota (Sevellec et al., 2018). There is increasing evidence that allochthonous microbial communities (hereafter the transient microbiota) ingested from the environment by the host play a significant role in the overall gut microbiota, either by stimulating colonization resistance or by providing additional functions to the host (e.g., David et al., 2014). However, few studies have tested for parallelism patterns in fish intestinal microbiota (Baldo et al., 2017; Baldo, Riera, Tooming‐Klunderud, Albà, & Salzburger, 2015; Hata et al., 2014; Sevellec et al., 2014; Smith, Snowberg, Caporaso, Knight, & Bolnick, 2015; Sullam et al., 2015). Also, the effect of the hybridization of two recently diverged species on microbiota composition is still poorly documented (Guivier et al., 2017).

The main goal of this study was to document the transient intestinal microbiota taxonomic composition of Lake Whitefish species pairs and their hybrids in natural and controlled environment. We investigated the transient intestinal microbiota in five wild species pairs of whitefish to estimate the within‐ and between‐lake variation and tested for parallelism among lakes. Secondly, we characterized the taxonomic composition of transient intestinal microbiota on dwarf, normal, and first‐generation hybrids reared in common garden in order to test the influence of the whitefish host on the microbiota in the same controlled conditions and under two different diets.

## 2 MATERIALS AND METHODS

### 2.1 Sample collection of wild whitefish

Lake Whitefish were sampled from May to July 2013 in Cliff, Indian, and Webster lakes in Maine, United States, and in East and Témiscouata lakes in Québec, Canada (Table 1). Fish were dissected in the field in sterile conditions as detailed previously (Sevellec et al., 2018). The intestine was cut at the hindgut end level (posterior part of the intestine), and the digesta were aseptically squeezed to collect the alimentary bolus. All samples of alimentary bolus were transported to the laboratory and kept at −80°C until further processing.

**Table 1:** Number and locations of samples, sampling dates for each captive and wild whitefish populations or group

Origin	Form	Sample size	Sampling date	Coordinates
Cliff	DD	12	13 to 14 June 2013	46°23′59″N, 69°15′11″W
	NN	12		
East	DD	10	2 to 4 July 2013	47°11′15″N, 69°33′41″W
	NN	13		
Indian	DD	12	10 to 11 June 2013	46°15′32″N, 69°17′29″W
	NN	13		
Témiscouata	DD	10	28 to 30 May 2013	47°40′04″N, 68°49′03″W
	NN	14		
Webster	DD	3	12 to 13 June 2013	46°09′23″N, 69°04′52″W
	NN	12		
Common Garden 1	DD	7	12 November 2013 to 09 June 2014	LARSA
	NN	5		
	DH	7		
	NH	6		
Common Garden 2	DD	5	12 November 2013 to 10 June 2014	LARSA
	NN	4		
	DH	6		
	NH	6		
Common Garden 3	DD	8	12 November 2013 to 11 June 2014	LARSA
	NN	6		
	DH	6		
	NH	8		

Abbreviations: DD, dwarf whitefish; DH, hybrid F1 D♀×N♂; NH, hybrid F1 N♀×D♂; NN, normal whitefish.

### 2.2 Experimental crosses, rearing conditions, and sample collection for captive whitefish

**Figure 1:**
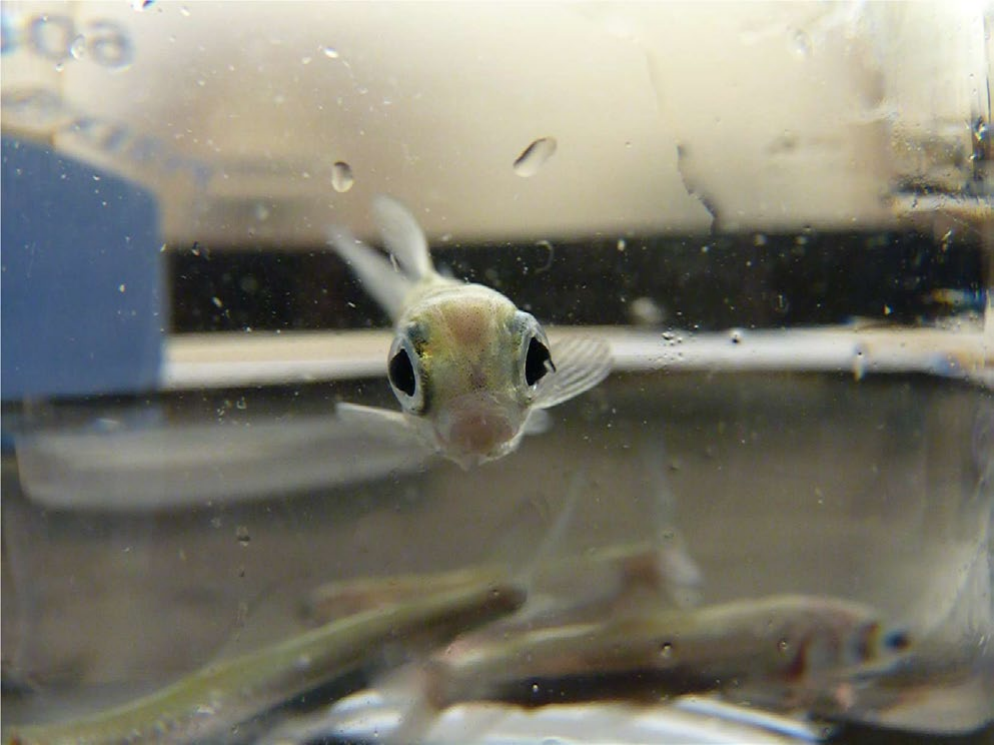
Picture of a juvenile captive hybrid whitefish at the beginning of experiment (November 2013)

In November 2013, 32 fish representing four cross types, dwarf (D♀×D♂), normal (N♀×N♂), and their reciprocal hybrids (F1 D♀×N♂ and F1 N♀×D♂), were pooled together in three tanks (eight fish/form/tank) (Figure 1). Experimental cross design was as described previously (Dalziel et al., 2015; Laporte et al., 2016). The protocol used for whitefish eggs fertilization and creating the parental generation is detailed in Appendix S1. Fish were separated in three tanks sharing the same experimental conditions (water, food, pH, and temperature) for seven months. Juvenile whitefish were fed on two types of food: *Artemia* and dry food pellet BioBrood (Bio‐Oregon^®^) (Flüchter, 1982; Zitzow & Millard, 1988). Fish were reassigned to their group of origin based on genetic allocation using mitochondrial and microsatellite DNA markers (Appendix S1). In June 2014, fish were euthanatized with MS‐222 and dissected immediately in sterile conditions, as described previously (Sevellec et al., 2018). Samples were kept at 80°C until further processing. This study was approved under Institutional Animal Care and Use Committee protocol 2008‐0106 at Laval University.

### 2.3 Whitefish microbiota: DNA extraction, amplification, and sequencing

The alimentary boluses of all fish were extracted using a modification of the QIAmp© Fast DNA stool mini kit (QIAGEN) (Appendix S1). In order to construct the community library, a region ~250 bp in the 16S rRNA gene, covering the V3‐V4 region, was amplified (detailed in Appendix S1) using specific primers with Illumina barcoded adapters Bakt_341F‐long and Bakt_805R‐long in a dual indexed PCR approach (Klindworth, Pruesse, & Schweer, 2012). All PCR results, including negative controls, were purified using the AMPure bead calibration method, quantified using a fluorometric kit (QuantIT PicoGreen; Invitrogen), pooled in equimolar amounts, and sequenced paired‐end using Illumina MiSeq at the Plate‐forme d'analyses génomiques (IBIS, Université Laval).

### 2.4 Amplicon analysis

Raw forward and reverse reads were quality trimmed, assembled into contigs for each sample, and classified using Mothur v.1.36.0 following the protocol of MiSeq SOP (https://www.mothur.org/wiki/MiSeq_SOP) (Kozich, Westcott, Baxter, Highlander, & Schloss, 2013; Schloss et al., 2009). Contigs were quality trimmed using several criteria. First, a maximum of two mismatches were allowed when aligning paired ends and ambiguous bases were excluded. Second, homo‐polymers of more than eight, sequences with lengths <400 bp and >450 bp, sequences from chloroplasts, mitochondria, and nonbacterial were removed. Thirdly, chimeric sequences were found and removed using the UCHIME algorithm (Edgar, Haas, Clemente, Quince, & Knight, 2011). Moreover, the database SILVA was used for the alignment and the database RDP (v9) was used to classify the sequences with a 0.03 cutoff level. The Good's coverage index, which was used to evaluate the quality of the sequencing depth, was estimated in Mothur (Hurlbert, 1971).

### 2.5 Statistical analyses

The analyses of microbiota were performed with Mothur and Rstudio v3.3.1 (RStudio Team, 2015). We first constructed a matrix of taxonomic composition (wild and captive included) with the number of operational taxonomic units (OTUs) after merging them by genus. The bacterial genera were considered as variables and fish as objects according to Mothur taxonomy files.

Details of the statistical analyses to test the effect of captivity (wild and captive conditions), the intestinal microbiota variation within and among wild whitefish populations as well as among the captive whitefish groups are presented in Appendix S1. In brief, a Spearman correlation matrix following a Hellinger transformation on the matrix of taxonomic composition was performed to document interactions between all captive and wild whitefish microbiota. The PERMANOVA analysis (number of permutations = 10,000) was also performed using the vegan package (Oksanen, Kindt, Legendre, & O'hara B., Stevens H.H., 2006) in R (Rstudio Team, 2015) on the matrix of taxonomic composition following a Hellinger transformation. An ANOVA following a fitted Gaussian family generalized model (GLM) was also performed at the alpha diversity level (inverse Simpson diversity) (Magurran, 2004). Furthermore, principal coordinates analyses (PCoAs) were built on a Bray–Curtis distance matrix after a Hellinger transformation to visualize variation between dwarf and normal whitefish within and among lakes (Legendre & Legendre, 1998; Oksanen et al., 2006). Finally, we documented the bacterial core of whitefish by identifying the bacterial genera present in 80% of all fish.

A linear discriminant analysis (LDA) was performed on the wild whitefish data, validated both according to (Evin et al., 2013) and from the PCA axes explaining at least 1% of the variation. The principal component analysis (PCA) was performed on the transformed Hellinger matrix.

In order to test for the presence of bacterial genera that were private to any of the captive whitefish group, we used the Metastats software with standard parameters (*p* ≤ .05 and number of permutations = 1,000) to detect differential abundance of bacteria at the genus level between two host populations (White, Nagarajan, & Pop, 2009). Four Metastats analyses were performed on the captive whitefish between: dwarf versus normal, dwarf versus hybrid F1 D♀N♂, normal versus hybrid F1 N♀D♂, and hybrid F1 D♀N♂ versus hybrid F1 N♀D♂.

## 3 RESULTS

### 3.1 Sequencing quality

A total of 2,498,271 sequences were obtained after trimming for the entire data set composed of 185 whitefish intestinal microbiota (67 dwarf whitefish, 79 normal whitefish, and 39 hybrids whitefish) from wild and captive populations (Table S1). A total of 189,683 OTUs were identified with a 97% identity threshold, representing 710 bacterial genera.

The average Good's coverage estimation for all intestinal microbiota (wild and captive whitefish) was 92.3 ± 7.6%. This apparently low Good's coverage essentially came from captive whitefish microbiota with a mixed diet of *Artemia* and dry food (*n* = 47), with a coverage index of 82.8 ± 3.4%. Indeed, the Good's coverage from wild whitefish microbiota (*n* = 111) and captive whitefish microbiota with a diet of *Artemia* only (*n* = 27) were, respectively, 95.4 ± 2.8% and 98.2 ± 1.4%, thus indicating a good sequencing quality of our data. These data were considered reliable for further analyses for three reasons. First, the mixed diet captive group was composed of 341 bacterial genera in which the distribution showed an unusual high abundance (i.e., number of reads) for a few genera (Table S2), which is known to decrease the Good's coverage which is defined as 1‐(Number of OTUs that have been sampled once/total number of sequences) (Hurlbert, 1971). Second, the Illumina MiSeq sequencing was performed in the same run for all samples, thus supporting the absence of sequencing problem given the excellent coverage obtained for the other groups. Third, a low Good's coverage is supposed to reflect a low number of sequences per sample because of the different filtration steps which eliminated reads generated by poor quality sequencing. Here, the low Good's coverage observed in the captive group that fed on a mixed diet showed a total number of sequences per sample similar to the other captive group (Table S2).

### 3.2 Wild versus captive whitefish intestinal microbiota

**Figure 2:**
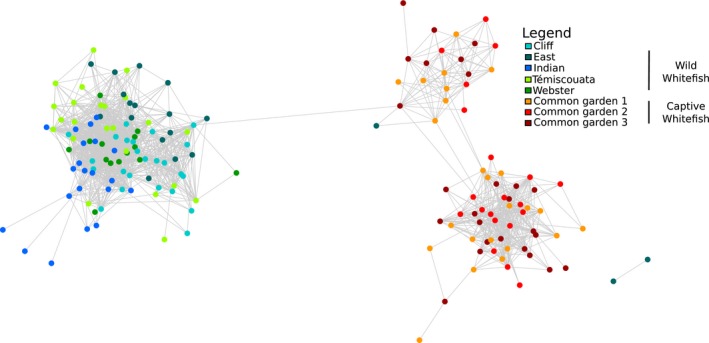
Network analysis of intestinal microbiota of dwarf and normal wild whitefish and intestinal microbiota of dwarf, normal, and hybrids captive whitefish. Each node represents either a dwarf, normal, or hybrid whitefish microbiota. The connecting lines between two samples represent their Spearman index correlation

**Figure 3:**
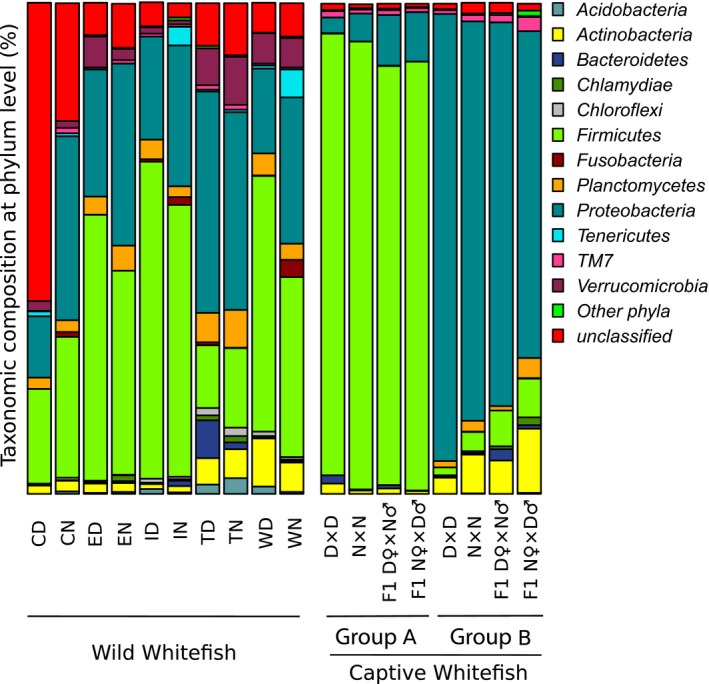
Relative abundance of phyla representatives found in intestinal microbiota for dwarf and normal wild whitefish in each lake, as well as in intestinal microbiota for dwarf, normal, and hybrids whitefish in controlled condition. Taxonomy was constructed with the database Silva and MOTHUR with confidence threshold at 97%. For the wild whitefish, lakes are represented as C: Cliff, E: East, I: Indian, T: Témiscouata, W: Webster, and the whitefish species is represented as D: dwarf and N: normal. For the captive fish, normal whitefish, dwarf whitefish, and hybrids are represented as N × N, D × D, F1 D♀×N♂ and F1 N♀×D♂, respectively. Diet group A (*Artemia* + dry food) and B (*Artemia*)

The network analysis among all samples revealed a pronounced differentiation in intestinal microbiota between wild and captive whitefish (Figure 2). More specifically, all wild whitefish was comprised in a first group except one dwarf and two normal all from East Lake. There was no clear pattern of differentiation between wild dwarf and normal whitefish microbiota (Figure S1) but all wild populations tended to cluster distinctively from captive fish. The second and third groups were composed by all captive whitefish with few interactions observed between them despite the fact that they both comprised fish from all four groups (dwarf, normal and both reciprocal hybrids). This second level of differentiation was based on diet variation between the two captive groups (Figure 2). The differentiation between the wild and the captive fish was also supported by a significant effect of captivity on taxonomic composition (PERMANOVA, *p* < .001; Table 2) when performing analysis using all fish, dwarf only, and normal only, as well as on alpha diversity when using all fish (ANOVA, *p* < .001; Table S3). Furthermore, although the major phyla (*Firmicutes*, *Proteobacteria*, *Actinobacteria*, and *Planctomycetes*) were similar between wild and captive whitefish, the bacterial abundance clearly differed between them (Figure 3). Finally, among the 710 bacterial genera found among all captive and wild whitefish, six were shared by all fish: *Acinetobacter*, *Aeromonas*, *Clostridium*, *Legionella*, *Methylobacterium*, and *Propionibacterium*. These constitute the core intestinal microbiota defined as the microbial component shared by 80% of the samples.

**Table 2:** Summary of PERMANOVA test statistics on microbiota taxonomic composition

Fish group	Source of variation	PERMANOVA		
		*F*‐value	*R* ^2^	*p*(>*F*)
Wild				
All lakes	Species	2.350	.017	.006
	Lake	6.744	.197	<.001
	Species:Lake	1.927	.056	<.001
	Body mass	1.628	.012	.067
Cliff Lake	Species	5.253	.180	<.001
	Body mass	2.914	.100	<.001
East Lake	Species	1.889	.085	.047
	Body mass	1.165	.053	.291
Indian Lake	Species	2.032	.083	.041
	Body mass	1.582	.064	.105
Témiscouata Lake	Species	0.741	.033	.732
	Body mass	0.920	.041	.447
Webster Lake	Species	0.858	.057	.562
	Body mass	2.142	.143	.015
Captive	Group	1.985	.043	
	Diet	58.955	.427	<.001
	Species:Diet	1.557	.034	.108
	Body mass	1.990	.014	.084
	Tank	1.649	.024	.102
Both				
All fish groups	Captivity	64.457	.260	<.001
	Body mass	3.481	.014	.001
Dwarf	Captivity	28.245	.289	<.001
	Body mass	4.517	.046	<.001
Normal	Captivity	16.371	.180	<.001
	Body mass	1.917	.021	.035

Note

First, the fish group “wild” refers to the analysis of effect of host species (dwarf and normal), lake (Cliff, East, Indian, Témiscouata, and Webster), and its interaction with body mass as a covariate on all wild fish. Second, the fish group “all lakes” tests the host species and body mass as a covariate is treated for each lake separately. Third, the fish group “captive” refers to the analysis of effect of host group (dwarf, normal, hybrids F1 D♀N♂, and F1 N♀D♂), diet (*Artemia* only and mixed diet of live *Artemia* with dry food), and its interaction with body mass and tank as covariates on all captive fish. Fourth, the fish group “both” refers to the effect of captivity (wild and captive) and body mass as covariate on all fish, dwarf only, and normal only. *F*‐value: value of the *F* statistic, *R* ^2^: *R*‐squared statistic, *p*(>*F*): *p*‐value. Only the interactions “Species:Lake” and “Species:Diet” are presented in this table.

### 3.3 Wild dwarf and normal whitefish microbiota

At the phylum level, dwarf and normal wild whitefish transient intestinal microbiota was characterized by identical dominant phyla with a similar bacterial abundance (Figure 3). However, variation in taxonomic composition between dwarf and normal whitefish was observed for less dominant phyla. For example, *Tenericutes* and *Fusobacteria* were more represented in normal, whereas *Bacteroidetes* was more represented in dwarf whitefish. We observed a more pronounced influence of the lake of origin on taxonomic composition whereby dwarf or normal microbiota within a given lake shared more similarities than microbiota from different lake populations within a same species (PERMANOVA_lake_, *p* < .001; PERMANOVA_species_ *p* < .006; Table 2) (Figure 3).

**Figure 4:**
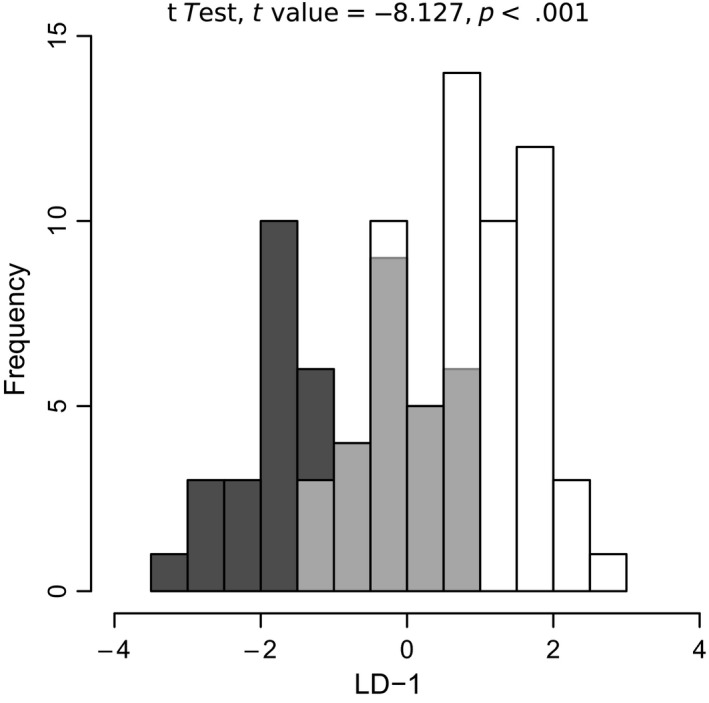
Linear discriminant analysis (LDA) histogram of all wild whitefish microbiota. This linear discriminant analysis was performed on the axes of principal component analysis (PCA) and *t* tests were performed on the results of the discriminant analysis. Dwarf and normal whitefish are represented by the black and white bars, respectively. Dwarf and normal whitefish with overlapping discriminant scores are shown in gray

**Figure 5:**
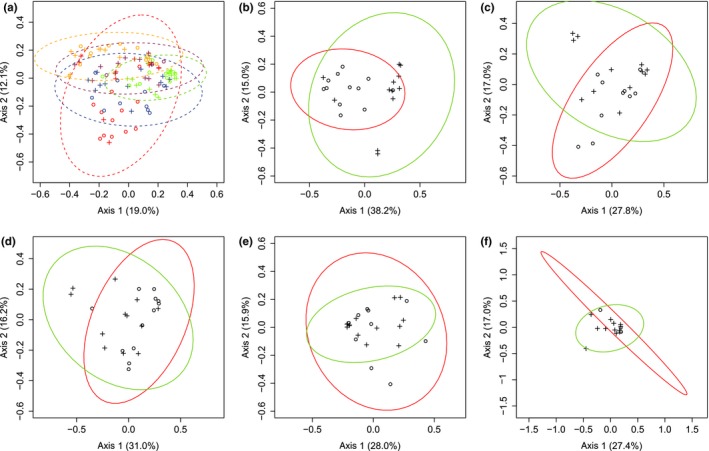
Principal coordinate analyses (PCoAs) within and between lakes for the wild whitefish microbiota. These PCoAs are based on Jaccard index after a Hellinger transformation. Ellipses of 95% confidence are illustrated and were done with dataEllips using R car package. (a) comparison among all wild whitefish populations from the five lakes. Each lake analyzed is represented by a different symbol and ellipse color: Cliff Lake (red), East Lake (blue), Indian Lake (orange), Témiscouata Lake (green), and Webster Lake (purple), and whitefish species is represented by symbols: Dwarf (circle) and Normal (cross). (b–f) comparison between Dwarf and Normal whitefish microbiota within each lake. Cliff Lake, East Lake, Indian Lake, Témiscouata Lake, and Webster Lake are represented by b, c, d, e, and f, respectively. Whitefish species is represented by different symbols: dwarf (circle) and normal (cross); ellipses of 95% confidence are illustrated and were done with dataEllips using R car package. The red and green ellipses represent the dwarf and normal species, respectively

Although no effect of lake or species on alpha diversity was observed (Table S3), there was a significant effect of both lake and host species on taxonomic composition (Table 2). The LDA performed on all wild whitefish also confirmed this overall difference between dwarf and normal intestinal microbiota albeit with overlap between them (Figure 4). Within each lake, the PERMANOVA revealed significant differences between dwarf and normal whitefish in three lakes (Cliff, East, and Indian lakes) but no difference in Témiscouata and Webster lakes (Table 2). Again, this suggested that the lake effect was more important than that of the host species. This was also supported by the PCoA analyses that revealed no global differentiation between all dwarf and normal whitefish (Figure 5a). Yet, host effect was supported in lake‐specific PCoAs based on partially overlapping 95% confidence interval in Cliff, East, and Indian lakes (Figure 5b,d). Complete overlap was observed in Témiscouata Lake (Figure 5e), whereas results were ambiguous in Webster Lake, most likely due to low sample size for this lake (Figure 5f).

### 3.4 Pure and hybrid whitefish microbiota in controlled environment

**Figure 6:**
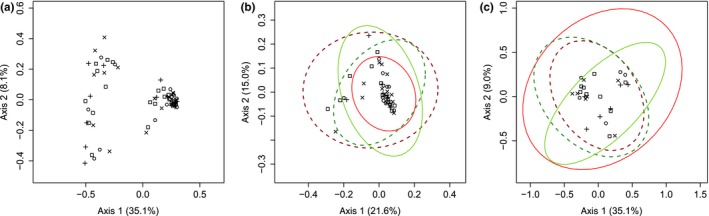
Principal coordinate analyses (PCoAs) between the microbiota of the four captive whitefish groups. (a) comparison between the four captive whitefish groups intestinal microbiota. (b) Comparison between the four whitefish groups intestinal microbiota in the mixed diet group. (c) Comparison between the four whitefish groups intestinal microbiota in the *Artemia* diet group. Ellipses of 95% confidence were done with dataEllips using R car package. Each whitefish species is represented by different symbols: dwarf (D♀×D♂), and normal (N♀×N♂) are represented by circle a cross respectively, and their ellipses are represented by continuous lines. The hybrid F1 N♀×D♂ and hybrid F1 D♀×N♂ are represented by the symbol × and □, respectively, and their ellipses are represented by dotted line. Dwarf and hybrid F1 D♀×N♂ are represented in red, whereas normal and hybrid F1 N♀×D♂ are represented in green

Although all fish were exposed to the same environment and the same food (both *Artemia* and dry fish food), we observed that some whitefish did not feed on the dry fish food and ate only live *Artemia*. As a result, we observed a mass and body length dichotomy between the two diet groups (Test of student, *p* < .001) (Table S4). As for the network analysis, the two distinct diet groups were evidenced by a significant effect of diet on both taxonomic composition microbiotas (PERMANOVA, *p* < .001; Table 2) and alpha diversity (ANOVA, *p* = .001; Table S3). The PCoA analysis clearly separated two distinct clusters on axis one corresponding to the two diet groups and independent of the genetic background (either pure forms or hybrids) (Figure 6). Furthermore, the mixed diet group was dominated by *Firmicutes* and the *Artemia* diet group was dominated by *Proteobacteria* (Figure 3). Within the mixed diet group, lower abundance for *Firmicutes*, but higher for *Proteobacteria,* was observed in reciprocal hybrids in comparison with dwarf and normal whitefish, whereas the opposite pattern was observed for the *Artemia* diet group (i.e., hybrids bacterial abundance was higher for *Firmicutes* but lower for *Proteobacteria*). Host group effect was also supported by the PERMANOVA (Table 2). The PCoA analysis within each of the two diet groups highlighted a modest differentiation based on overlapping 95% confidence interval between hybrids and pure whitefish (Figure 6). In the mixed diet group, dwarf and normal ellipses were mostly aligned on the second axis, whereas the ellipses of the two hybrid groups were mostly aligned on the first axis. The inverse pattern was observed in the *Artemia* diet group with the ellipses of the pure whitefish those of hybrid whitefish aligned on the first and second axes, respectively.

**Figure 7:**
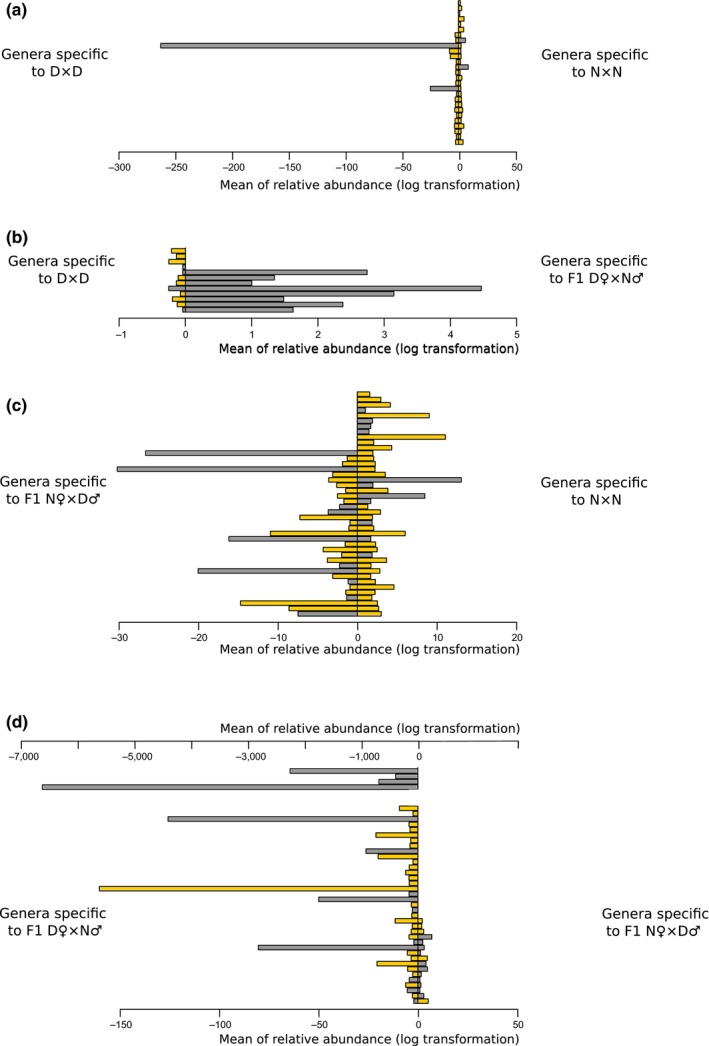
Metastats results for dwarf, normal, and hybrid captive whitefish. Four side‐by‐side comparisons were performed with dwarf (D♀×D♂), normal (N♀×N♂), hybrid F1 N♀×D♂, and hybrid F1 D♀×N♂. Each genus specific to a given whitefish group is represented by a bar plot. The abscissa represented the mean of the relative abundance of a genus specific after a log transformation. Mixed diet and *Artemia* diet groups are represented by yellow and gray bars, respectively

Between eight and 42 bacterial genera were differentially represented to a given whitefish group within diet groups (Figure 7). We observed 21 dwarf‐specific and 27 normal‐specific bacterial genera, respectively, whereas the comparison between hybrids F1 D♀N♂ and F1 N♀D♂ revealed 41 and 16 specific bacterial genera, respectively. Finally, we observed 135 specific bacteria genera in the mixed diet group versus 62 in the *Artemia* diet group (see Table S5 for details).

## 4 DISCUSSION

### 4.1 The intestinal microbiota of captive versus wild whitefish

Although an important part of bacteria which colonizes fish intestine may represent a random sampling from water and food, the occurrence of intestinal microbiota cores has been increasingly documented (Astudillo‐García et al., 2017). The intestinal microbiota cores represent OTUs or genera shared among closed host relatives. Thus, despite the fact that wild and captive whitefish studied here never shared a common environment (they grew in totally different waters), the comparison of their microbiota highlighted six genera shared by at least 80% of all samples. Interestingly, our intestinal core microbiota data represented 20% of shared sequences which is higher than the intestinal microbiota core reported for cichlid species (13%–15%) (Baldo et al., 2015). These shared genera could be horizontally transmitted and/or selected as a common set of bacteria (Baldo et al., 2015; Rawls, Mahowald, Ley, & Gordon, 2006). Although the captive whitefish were hatched in captivity, their parents were of wild origin. Therefore, the conservation of certain genera by many captive whitefish might corroborate the microbiota vertical transmission in fish. It is also noteworthy that we found many bacteria of unknown taxonomy (see Figure 3) and much more so in wild than in captive whitefish. This, along with previous studies emphasizes the fact that a considerable number of bacteria are waiting to be discovered in natural freshwater ecosystems.

### 4.2 No clear pattern of parallel evolution in transient intestinal microbiota between dwarf and normal whitefish in the wild

Parallelism refers to the evolution of similar phenotypic traits in independent populations (Schluter & Nagel, 1995) and has been well documented in several sympatric species throughout the north hemisphere, including in Lake Whitefish (Bernatchez et al., 2010; Østbye et al., 2006; Schluter, 2000). Given the difference in trophic and ecologic niches occupied by both species (Landry & Bernatchez, 2010; Landry, Vincent, & Bernatchez, 2007), we predicted that some level of parallelism in transient intestinal microbiota would be observed between dwarf and normal whitefish species pairs. The dwarf whitefish is a limnetic fish feeding on zooplankton, whereas the normal whitefish is a benthic fish feeding on zoobenthos and molluscs (Bernatchez, Chouinard, & Lu, 1999; Bodaly, 1979). Therefore, we expected that a different diet should bring the dwarf and normal whitefish of a given sympatric pair in contact with different bacterial communities, leading to a distinct transient intestinal microbiota in a similar manner in the different lakes. Indeed, differentiation of microbiota composition correlated with diet was previously observed (David, Veena, & Kumaresan, 2016; Haygood & Jha, 2016; Koo et al., 2017; Nayak, 2010; Zarkasi et al., 2016). Thus, the use of novel diet elements may produce a change in the microbiota composition by increasing or decreasing different bacterial strain according to their metabolic potential (Rosenberg & Zilber, 2013). This is also supported by the microbiota composition differentiation of the two diet groups observed in captivity in this study. Despite a global effect of species host on microbiota, we did not observe a clear pattern of parallelism among the five lakes comprising sympatric whitefish pairs studied here. Indeed, nonparallel difference between dwarf and normal whitefish microbiota composition was observed in three of the five lakes, whereas no difference was observed in the other two lakes. This indicated that the environment has a more pronounced effect than the species host on the transient intestinal microbiota of dwarf and normal whitefish. These results are in line with those obtained in a previous study in the same system but investigating kidney microbiota. Thus, Sevellec et al. (2014) showed that differences in bacteria composition between dwarf and normal whitefish were not parallel among lakes. However, unlike this study and in accordance with the higher diversity of prey types, normal whitefish kidney tissue consistently had a more diverse bacterial community and this pattern was parallel among lakes. Together, these results on whitefish microbiota add to building evidence from previous studies on this system that the adaptive divergence of dwarf and normal whitefish has been driven by both parallel and nonparallel ecological conditions across lakes, a situation reported in several other fishes (Oke, Rolshausen, LeBlond, & Hendry, 2017). Moreover, the water bacterial community of the same studied lakes was investigated previously and we found that each lake is characterized by a specific water bacterial community (Sevellec et al., 2018). This may reflect the differences in both biotic and abiotic factors among these lakes (Landry & Bernatchez, 2010; Landry et al., 2007). For instance, Cliff, Webster, and Indian lakes are characterized by a greater oxygen depletion and a lower zooplankton biomass, whereas East and Témiscouata lakes are characterized by more favorable environmental conditions with a more important biomass and broader size distribution of zooplanktonic prey and well‐oxygenated water (Landry et al., 2007). Therefore, the variation in water bacterial community along with the biotic and abiotic factors could underlie the more important lake effect than species host effect observed in the transient intestinal microbiota. Nevertheless, highly distinct bacterial composition between the water bacterial community and the whitefish transient intestinal microbiota was observed among lakes. The water bacterial community was dominated by *Proteobacteria*, *Actinobacteria,* and *Bacteroidetes,* whereas the whitefish transient intestinal microbiota was dominated by *Firmicutes* and *Proteobacteria* (Sevellec et al., 2018). Therefore, whitefish transient intestinal microbiota was not directly reflective of its local environment, which raises the hypothesis of a selective effect on microbiota induced by host physiology, immunity, and genetic background (Alberdi et al., 2016; Macke et al., 2017). For instance, some transient bacteria might contribute to digestion of host diet (Smith et al., 2015) and, in turn, may impact on the transient intestinal microbiota composition by increasing their abundance (Rosenberg & Zilber, 2013).

### 4.3 Comparison of transient and adherent intestinal microbiota in wild whitefish and the host effect

The most prevalent phyla in wild whitefish transient microbiota are *Acidobacteria, Actinobacteria, Bacteroidetes, Chlamydiae, Chloroflexi, Firmicutes, Fusobacteria, Planctomycetes, Proteobacteria, Terenicutes, TM7,* and *Verrucomicrobia*, which have also been reported in previous studies of freshwater fishes (Eichmiller, Hamilton, Staley, Sadowsky, & Sorensen, 2016; Larsen & Mohammed, 2014; Li, Zhu, Yan, Ringø, & Yang, 2014; Roeselers et al., 2011; Sullam et al., 2012; Ye, Amberg, Chapman, Gaikowski, & Liu, 2014). In a previous study on adherent intestinal microbiota (that is adherent to the intestinal mucosa) performed on the same individuals, we found that while adherent and transient intestinal were characterized by similar major phyla, the abundance of some of them was different (Sevellec et al., 2018). For example, the five first phyla for the adherent microbiota were *Proteobacteria* (39.8%), *Firmicutes* (19%), *Actinobacteria* (5.1%), *OD1* (3.8%), and *Bacteroidetes* (2.8%), whereas the first five phyla for the transient microbiota were *Firmicutes* (38.2%), *Proteobacteria* (29.5%), *Verrucomicrobia* (4.4%), *Planctomycetes* (4.1%), and *Actinobacteria* (3.7%). Moreover, the number of genera and the number of OTUs were about 50% more important in the transient microbiota (611 genera and 94,883 OTUs) than the adherent microbiota (421 genera and 10,324 OTUs). Most of the adherent bacterial taxa living on the intestinal mucosa are not randomly acquired from the environment (Bolnick et al., 2014), but are rather retained by different host characteristics (Brucker & Bordenstein, 2012). Similarly, we previously reported that there is an important host effect in both dwarf and normal whitefish, which stabilizes the number of bacterial genera living in the intestinal mucosa (Sevellec et al., 2018). Thus, the comparison between whitefish transient and adherent microbiota supports the view that the whitefish host have a selective effect on its intestinal microbiota. For instance, dwarf and normal whitefish in Cliff and East lakes show a distinct intestinal microbiota for both the adherent and the transient bacteria, whereas the adherent, but not the transient intestinal microbiota differed between species in Témiscouata Lake, and the opposite was observed in Indian Lake. In Témiscouata Lake, this difference in adherent microbiota between species suggested a host species effect leading to differential abundance of the same bacterial taxa. In contrast, results in Indian Lake suggest that host species have no clear effect on microbiota divergence and that the difference in transient microbiota is likely caused by the trophic niches occupied by each species. Altogether, these observations suggest that the direction and intensity of factors determining the composition of intestinal microbiota may differ between the host and the microbiota of a given holobiont system, as previously reported (Rosenberg & Zilber, 2016). Here, we tentatively propose that three putative distinct host–microbiota interactions may have evolved independently in postglacial time: (a) divergence of intestinal microbiota influenced by the host and the environment (Cliff and East lakes), (b) divergence of the intestinal microbiota mostly influenced by the host (Témiscouata Lake), and (c) divergence of intestinal microbiota mostly influenced by the environment (Indian Lake). While speculative at this point, these putative distinct host–microbiota interactions would deserve to be carefully evaluated in future host–microbiota studies in a speciation context. Finally, given the pronounced difference that may exist between transient and adherent microbiota, our results suggest that adherent microbiota is a more reliable choice to study the effect of host species on microbiota than the analysis of transient microbiota.

### 4.4 Modest but significant host effect on the transient intestinal microbiota in controlled conditions

An unplanned variation in our experimental set up occurred during the captive rearing of the whitefish pair species and the reciprocal hybrids for seven months, which led to the unexpected observation of a diet preference which split the captive whitefish into two groups independently of the parental or hybrid origin or the tanks where fish were. The use of two types of food, *Artemia* and dry pellets, is usually recommended for optimizing growth and survival of juvenile whitefish in captivity (Flüchter, 1982; Zitzow & Millard, 1988). However, while 47 whitefish opted to feed on both types of diet, 27 chose to feed only on *Artemia*. Indeed, *Artemia* as the only source of food cannot provide the good nutrients used for the juvenile whitefish growth (Zitzow & Millard, 1988). As a result, all normal length whitefish were in the group A (dry food and *Artemia*), whereas all the small length whitefish were in the group B (*Artemia* only). (Table S4). We believe that it is very unlikely that any factor other than different diet would have caused such a strong association between size and microbiota. Indeed, this allowed us to assess the impact of different diets in an otherwise identical controlled environment, which revealed that diet had the most profound impact on the community composition of transient intestinal microbiota in a controlled environment.

Nevertheless, we did observe a significant, albeit modest effect of host groups on the transient intestinal microbiota. In principle, in a controlled environment, there should be no environmental effect on the microbiota composition, and consequently, variation in microbiota should only depend on the host effect which integrated the influence of the host physiology, immunity, and genetic background. Here, while the PCoA analysis only revealed a slight pattern of differentiation between both parental species and their reciprocal hybrids, the PERMANOVA test revealed a statistically significant effect of the host genetic background on the taxonomic composition of the transient microbiota. This was accompanied by a significant variation in bacterial abundance at the phylum level, especially within the mixed diet group feeding on both *Artemia* and dry pellets. Finally, numerous genera that were specific to one whitefish species or the hybrids were observed in both diet groups. These results suggest an effect of hybrid genetic background on the transient intestinal microbiota. This effect could hypothetically be explained Bateson, Dobzhansky, and Muller (BDM) genetic incompatibilities previously documented in whitefish (Dion‐Cote, Renaut, Normandeau, & Bernatchez, 2014; Gagnaire et al., 2013; Renaut, Nolte, & Bernatchez, 2009). To our knowledge, only one study compared the intestinal microbiota among closely related fish populations in controlled conditions (Sullam et al., 2015) and none compared parental and hybrid progeny. Specifically, distinct intestinal microbiota between two ecotypes of the Trinidadian Guppy (*Poecilia reticulata*) suggested a pronounced effect of the genetic background (Sullam et al., 2015). However, these results should be interpreted cautiously since fish used for this experiment were adults that were born in the wild and kept in tanks for 10 weeks only. Consequently, the difference could reflect a carry‐over effect from the natural conditions, whereas in our case, fish were born in captivity.

To conclude, our results show that the transient intestinal fish microbiota is the result of complex interactions between the host's genetic background and environmental conditions. The prevalent environmental effect on the microbiota we observed among five sympatric whitefish pairs in the wild illustrates that drawing generalization regarding host–microbiota association for a given species may be difficult, and in fact inappropriate.

## CONFLICT OF INTEREST

The authors declare no conflicts of interest.

## AUTHOR CONTRIBUTIONS

MS and LB conceived the study. LB contributed resources. MS and ML collected samples and analyzed the data. AB and MS performed laboratory assays. ND provided bioinformatics support (network analyses). MS performed bioinformatic work and wrote the manuscript, while LB, ML, and ND helped to draft and improve the manuscript. All authors edited the manuscript and approved the final version.

## Data Availability

Sequencing results are available in the Sequence Read Archive (SRA) database at NCBI under BioProject ID SUB3062520.
