# Supplementary figures and images for: Evidence for host effect on the intestinal microbiota of whitefish (Coregonus sp.) species pairs and their hybrids
Source: Ecol Evol. 2019 Oct 2;9(20):11762–74. doi: 10.1002/ece3.5676 (PMC6822036; doi:10.1002/ece3.5676)

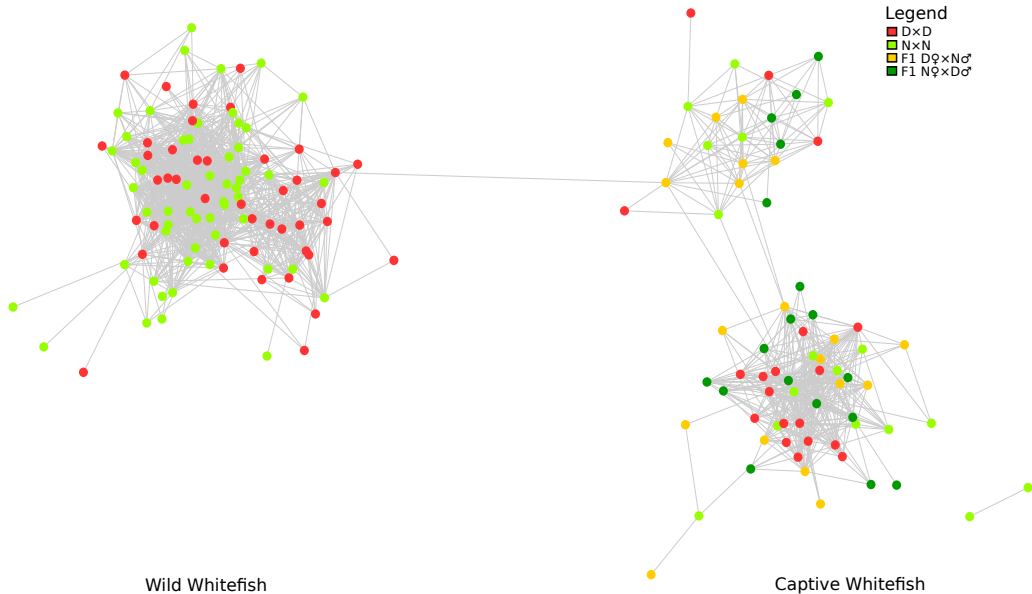

Supplement: Supplementary file 1 [file ECE3-9-11762-s001.pdf]
