# Supplementary material for: Evidence for host effect on the intestinal microbiota of whitefish (Coregonus sp.) species pairs and their hybrids
Source: Ecol Evol. 2019 Oct 2;9(20):11762–74. doi: 10.1002/ece3.5676 (PMC6822036; doi:10.1002/ece3.5676)
Supplement: Supplementary file 7 [file ECE3-9-11762-s007.docx]

**Appendix S1**

### Experimental crosses of captive whitefish

### Whitefish eggs used for this study were incubated at the Laboratoire de Recherche en Sciences Aquatiques (LARSA, Université Laval, Québec, Canada). The dwarf species came from Témiscouata Lake (47°40’04”N, 68°49’03”W) which is from the Acadian glacial lineage origin whereas the normal species came from Aylmer Lake (45°54”N, 71°20”W) corresponding to the Atlantic glacial lineage (Bernatchez & Dodson, 1991). Backcross F1-Hybrids were obtained by crossing a F1 hybrids laboratory strain and wild whitefish parents. More precisely, F1 hybrid (F1 D♀*N♂) were produced in crossing three wild dwarf females and two laboratory strain normal males (born in laboratory from Aylmer Lake population origin) by artificial fertilization. Same processes was used to produced F1 hybrid (F1 N♀*D♂) with crossing five laboratory strain normal females and twelve wild dwarf males (see figure 1 (Rogers et al., 2007)). The dwarf and normal whitefish crosses were also created by artificial fertilization with sperm and eggs were collected in the field and transported to the LARSA. No treatments, such as antibiotics or malachite green were delivered to the eggs.

### Whitefish microbiota: DNA extraction, amplification and sequencing

Slight modifications of the QIAmp© Fast DNA stool mini kit (QIAGEN) were performed to extract bacterial DNA captive and wild fish intestinal bolus. To maximize DNA extraction of gram-positive bacteria, temperature and time were increased during the incubation steps and all products used were doubled (Proteinase K, Buffer AL and ethanol 100%). Thus, 1200 µl were transferred into the column (in two subsequent steps) and bacterial DNA was eluted from the column with 100 μl of ultrapure water (DEPC-treated Water Ambion®). DNA extractions were quantiﬁed with a Nanodrop (Thermo Scientiﬁc) and stored at −20°C until use. Five blank extractions were also done as negative controls.

The PCR amplification comprised 50 µl PCR amplification mix containing 25 µl of NEBNext Q5 Hot Start Hifi PCR Master Mix, 1 µl (0.2 µm) of each speciﬁc primers (Bakt_341F-long and Bakt_805R-long), 15 µl of sterile nuclease-free water and 8µl of specify amount of DNA. The PCR program consisted of an initial denaturation step at 98°C for 30s, followed by 30 cycles, where one cycle consisted of 98°C for 10 s (denaturation), 56°C for 30 s (annealing) and 72°C for 45s (extension), and a final extension of 72°C for 5 min. Negative and positive controls were also performed using the same program.

**Statistical analyses**

We first investigated the microbiota difference between the captive and wild whitefish using a network analysis. A Spearman’s correlation matrix following a Hellinger transformation on the matrix of taxonomic composition was performed to document interactions between all captive and wild whitefish microbiota. More precisely, a Spearman’s correlation value (threshold ≥0.5), a P-value and Bonferroni correction was calculated for each sample. The network was visualized using Cytoscape v3.2.1 (Shannon *et al.*, 2003), where nodes were illustrated in two different versions: (i) according to their sampling sites (eight groups: five lakes and three tanks) and (ii) according to their genetic group (the two wild species pairs and the four captive groups: dwarf, normal, reciprocal hybrid F1 D♀N♂, and hybrid F1 N♀D♂). We also tested for the effect of captivity (wild and captive conditions) on whitefish microbiota taxonomic composition (PERMANOVAs; 10,000 permutations) and alpha diversity (inverse Simpson diversity) with an ANOVA following a fitted Gaussian family generalized model (GLM) (Magurran, 2004). This was performed on all fish, on dwarf whitefish only and on normal whitefish only.

Secondly, in order to document variation within and among wild whitefish populations, we tested for an effect of ‘host species’, ‘lake’ and their interaction, with ‘body mass’ as a covariate on the taxonomic composition, using a permutational analysis of variance (PERMANOVA; 10,000 permutations). This procedure was run for each of the five lakes independently after removing the explanatory variable ‘lake’ of the analysis. The ‘host species’, ‘lake’ effects and their interaction on the inverse Simpson diversity were also tested using an analysis of variance (ANOVA) following a fitted Gaussian family generalized model (GLM). Allometric effect on inverse Simpson diversity was first tested with a linear regression on body mass. As for the taxonomic composition, we ran this procedure for each lake independently. Furthermore, principal coordinates analyses (PCoAs) was built on a Bray-Curtis distance matrix after a Hellinger transformation to visualize variation at the genus level between dwarf and normal wild whitefish among and within the lakes (Oksanen *et al.*, 2006; Legendre & Legendre, 1998).

Finally, we tested for differences in taxonomic composition between the four captive groups by investigating the effect of ‘host group’ (Dwarf, Normal, hybrid F1 D♀N♂, and hybrid F1 N♀D♂), ‘diet’ and their interaction with ‘body mass’ and ‘tank’ as covariates (PERMANOVA; 10,000 permutations). The effect of diet was added in the analysis because fish bolus exhibited a clear distinction between two observed feeding habits during the controlled experiment (A: feeding on a mix of dry food and *Artemia*, B: feeding on *Artemia* only). For the alpha diversity, the effect of ‘host group’, ‘diet’ and their interaction on the inverse Simpson diversity were tested with a mixed effects linear random model using the ‘nlme’ package in R, with tank as a random effect and individual fish nested within tank (Pinheiro *et al.*, 2009). As for the analyses on wild whitefish, we first tested for an allometric relationship with body mass using a linear regression and used the residuals in all cases showing a significant relationship. Principal coordinates analyses (PCoAs) built on a Bray-Curtis distance matrix after a Hellinger transformation were also used to visualize variation at the genus level as described above. Linear discriminant analyses were also performed on captive whitefish but results were not displayed because of a negative cross-validation according to Evin *et al.*(2013).

### Whitefish host: DNA extraction, amplification and genetic identification of captive whitefish lineages

A fin clip was collected from all fish and DNA was extracted using a salt extraction method (Aljanabi & Martinez, 1997) with slight modifications (Valiquette *et al.*, 2014). Mitochondrial (mtDNA) and nuclear DNA were used to identify the whitefish dwarf and normal, and their hybrids (F1 hybrid D♀N♂ and F1 hybrid N♀D♂). First, an analysis of mtDNA restriction fragment length polymorphism (RFLP) was performed as described in Dalziel et al. since pure dwarf and normal species possess distinct mitochondrial DNA haplotypes (Jacobsen *et al.*, 2012; Dalziel *et al.*, 2015). In brief, after the amplification of the cytochrome b by PCR, the amplified products were digested with SnaBI which cuts the amplified cytochrome b of the normal whitefish haplotype but not of the dwarf. Second, 12 nuclear microsatellite loci were genotyped on all juvenile whitefish and their known parents to differentiate them at the nuclear DNA level and details about primer sequences and PCR protocols are presented in Rico et al.. Three different PCRs were performed for this whitefish microsatellite markers analysis (Rico *et al.*, 2013). Firstly, the multiplex PCR A was performed with 2 µl (≈20 ng) of whitefish DNA, 5 μL Qiagen® multiplex reaction buffer, forward and reverse primers at different concentrations: 0.3 µm of Cocl32, Cocl lav41, Cocl Lav8 and 0.35 µm of Cocl Lav224; purified water adjusted the final volume at 10 µl. Multiplex PCR program was: 15 min at 94°C, and then 35 cycles of 30 sec at 94°C, 3 min at 58°C, 1 min at 72°C and 30 min at 60°C. Secondly, the multiplex PCR B were performed with 2 µl (≈20 ng) of whitefish DNA, 5 μL Qiagen® multiplex reaction buffer and forward and reverse primers at different concentration: 6 µm of Cocl15 et Cisco200 and 0.25 µm of Cocl 33; purified water adjusted the final volume at 10 µl. Multiplex PCR program was: 15 min at 94°C, and then 35 cycles of 30 sec at 94°C, 3 min at 60°C, 1 min at 72°C and 30 min at 60°C. Thirdly, the Simplex PCRs were performed with 2 µl (≈20 ng) whitefish DNA, 0.2 µl GoTaq® DNA polymerase (PROMEGA), 0.5 µl of each forward and reverse markers (0.5 µm) (Osmo5, Cocl34, Cocl36, Bwf F-1 and Cocl Lav22) 2 µl of 5X Colorless GoTaq®, 0.6 µl of MgCl2 (0.5 mM), 0.8 µl dNTPs (200 µm) and purified water adjusted the final volume at 10 µl. Simplex PCR program was: 2 min at 94°C, and then 35 cycles of 30 sec at 94°C, 3 min at 58°C (Osmo5, Cocl36, Bwf F-1, Cocl Lav22) or 64°C (Cocl34), 1 min at 72°C and 30 min at 60°C. Amplified loci were migrated via electrophoresis using an ABI 3130xl capillary DNA sequencer (Applied Biosystems Inc.) with a molecular size standard (GeneScan-500 LIZ, Applied Biosystems). Genotypes were scored using Genemapper 4.0 (Applied Biosystems Inc). A combination of three software, STRUCTURE v2.3.4, GENECLASS2 v2.0 and PAPA v2.0 was used to reassign each studied fish to its group of origin (Pritchard *et al.*, 2000; Duchesne *et al.*, 2002; Piry *et al.*, 2004). STRUCTURE was performed assuming an admixture model without priors with a burn-in period of 50 000 followed and 100,000 Markov Chain Monte Carlo (MCMC) steps. GENECLASS2 was conducted using the simulation test of (Rannala & Mountain, 1997) based on 100,000 simulated individuals. Finally, PAPA was performed for the parental allocation procedure with a uniform error model (error sum = 0.02).

**Supplementary Figures**

Figure S1 Network analysis of intestinal microbiota of dwarf and normal wild whitefish and intestinal microbiota of dwarf, normal and hybrids captive whitefish. The nodes represent a dwarf or a normal or a hybrid whitefish microbiota. More precisely, DD: dwarf whiteﬁsh, NN: normal whiteﬁsh, DH: hybrid F1 D♀*N♂, NH: F1 N♀*D♂. The connecting lines between two samples represent their correlation and is highlighting by a Spearman index.
